# Early Improvement in Psychosocial Function Predicts Longer-Term Symptomatic Remission in Depressed Patients

**DOI:** 10.1371/journal.pone.0167901

**Published:** 2016-12-28

**Authors:** Manish K. Jha, Abu Minhajuddin, Tracy L. Greer, Thomas Carmody, Augustus John Rush, Madhukar H. Trivedi

**Affiliations:** 1 Department of Psychiatry, UT Southwestern Medical Center, Dallas, Texas, United States of America; 2 Department of Clinical Sciences, UT Southwestern Medical Center, Dallas, Texas, United States of America; 3 Department of Psychiatry and Behavioral Sciences, Duke-National University of Singapore, Singapore; Nagoya Mental Clinic, JAPAN

## Abstract

The goal of this study was to evaluate the relationship between early change in psychosocial function independent of depression severity and longer-term symptomatic remission. Participants of Combining Medications to Enhance Depression Outcomes trial were randomly selected for model selection (n = 334) and validation (n = 331). Changes in psychosocial function (Work and Social Adjustment Scale, WSAS) from baseline to week 6 were assessed and two data-driven sub-groups of WSAS change were identified in the randomly selected model selection half. Results of analyses to predict symptomatic remission at 3 and 7 months were validated for these sub-groups in the second half (validation sample). From baseline to week 6, psychosocial function improved significantly even after adjusting for depression severity at each visit and select baseline variables (age, gender, race, ethnicity, education, income, employment, depression onset before age 18, anxious features, and suicidal ideation), treatment-arm, and WSAS score. The WSAS change patterns identified two (early improvement and gradual change) subgroups. After adjusting for baseline variables and remission status at week 6, participants with early improvement in the second half (validation sample) had greater remission rates than those with gradual change at both 3 (3.3 times) and 7 months (2.3 times) following acute treatment initiation. In conclusion, early improvement in psychosocial function provides a clinically meaningful prediction of longer-term symptomatic remission, independent of depression symptom severity.

## Introduction

Treatment algorithms largely rely on depressive symptom severity as to inform clinical decisions [1–4], even though symptom severity reflects only a portion of the burden of Major Depressive Disorder (MDD) [5]. Despite its importance to depressed patients as a primary outcome, psychosocial function has received far less attention in either practice or treatment research [6]. Although it is significantly impaired in depressed patients and improves substantially with effective antidepressant treatments [7–13], psychosocial function has been traditionally considered a lagging indicator of improvement rather than a critical domain of interest and treatment focus, apparently due to smaller magnitude and slower onset of change when compared to reduction in depression severity [7, 11, 14–17]. The importance of psychosocial function in patient care is highlighted by evidence that psychosocial impairments often persist after symptomatic remission [18], are associated with worse long-term clinical outcomes [19–21], and remain a major concern for patients [22]. Functional recovery is distinct from symptomatic recovery and likely requires different treatment approaches [23, 24].

To identify those patients in need of additional efforts to improve psychosocial function, assessment of function should be distinct and not simply reflect depressive symptom severity. Patterns of improvement with antidepressant treatment may further help in targeting adjunctive treatments needed to attain functional recovery. Using latent class analysis, Uher et al. [2010] previously found that sub-groups of depressed patients had two distinct trajectories (rapid-initial and gradual) of depressive symptom improvement [25] with potential treatment implications; patients on a noradrenergic antidepressant (nortriptyline) were more likely to display rapid-initial improvement as compared to those on a serotonergic antidepressant (escitalopram) [26]. Hence, this data-driven approach can be extended to identify sub-groups of patients with different trajectories of change in psychosocial function early in the course of antidepressant treatment. We have found that work productivity (absence from work and/or reduced productivity while at work) improvements early in the course of antidepressant treatment were not only independent of change in depression severity, but patterns of this improvement could also be used to predict long-term clinical outcomes [27]. These findings are applicable only to employed persons

Previous reports based on findings from data-driven model-based approaches have significant limitations because findings are typically specific to the sample in which they are developed and replication in separate sample(s) have been rare [27]. Even beyond the need to replicate model-based analyses, there has been a growing appreciation of the need for reproducible published research reports [28]. In a recent report that replicated 97 previously published psychology studies with positive findings, only a third of the replications were positive [29]. While neuroimaging studies routinely incorporate replication paradigms [30], to our knowledge no previous study has tried to replicate findings of data-driven latent class (trajectory) analyses for changes on psychosocial function.

This study evaluates whether changes in psychosocial function that occur early (within the first 6 weeks) in the course of antidepressant treatment, independent of change in depression severity, and can be used to predict future clinical course. All participants (n = 665) of the Combining Medications to Enhance Depression Outcomes (CO-MED) trial were divided randomly in two halves, model selection (n = 334) and validation (n = 331) samples, to generate two separate non-overlapping groups so that inferences can be drawn on model selection sample and reproduced in the validation sample. In the model selection sample, model-based latent class analyses were used to identify sub-groups of participants with different trajectories of change in psychosocial function. In the validation sample, model parameters from the model selection sample were used to assign sub-groups of changes in psychosocial function. Improvement in psychosocial function and differential rates of remission at 3 and 7 months based on sub-groups of changes in psychosocial function were tested.

## Methods

### Study overview

Data for this report were obtained from the CO-MED trial as an unplanned secondary analysis [9]. This study recruited 665 participants who were stratified by clinical sites and assigned to one of the treatment-arms in a 1:1:1 ratio. The three treatment-arms in this study were: 1) escitalopram plus placebo (selective serotonin reuptake inhibitor [SSRI] monotherapy), 2) sustained-release (SR) bupropion plus escitalopram (bupropion-SSRI combination), and 3) extended-release (XR) venlafaxine plus mirtazapine (venlafaxine-mirtazapine combination).

Study visits were conducted at baseline and weeks 1, 2, 4, 6, 8, 10, and 12 for acute-phase and weeks 16, 20, 24, and 28 for continuation-phase. Participation in continuation-phase treatment (after 3 months) was restricted to participants who had either 1) received an acceptable benefit by 3 months as defined by score of 9 or less on 16-item Quick Inventory of Depressive Symptomatology–Clinician rated (QIDS-C) [31] or 2) had reached a score of 10–13 on QIDS-C and both physician and participant decided to continue treatment because of substantial benefit to study medications. At each visit, study physicians used Measurement Based Care (MBC) (1) to personally tailor dosage adjustments based on QIDS-C scores and scores on the Frequency, Intensity, and Burden of Side Effects Rating (FIBSER) scale [32]. The QIDS-C items were extracted from the Inventory of Depressive Symptomatology–Clinician-Rated (IDS-C) scale [33].

### Participants

Broad inclusion and minimal exclusion criteria were used to ensure a reasonably representative participant group [9]. Patients seeking treatment at participating clinical sites and planning to continue living in the area of that clinical site for the duration of the study were eligible for enrollment. Clinical sites included six primary and nine psychiatric care clinics across the United States (full list available at https://clinicaltrials.gov/ct2/show/NCT00590863) that were selected based on previous performance in the Sequenced Treatment Alternatives to Relieve Depression trial in order to ensure adequate minority representation. To be included in the study, participants had to meet clinical criteria for nonpsychotic MDD, recurrent (greater than 1 previous episode) or chronic (current episode greater than 2 years), as defined by a clinical interview and confirmed by the MINI International Neuropsychiatric Interview (MINI) [34]; have at least 2 months’ duration of current episode; and score 16 or greater on the 17-item Hamilton Rating Scale for Depression (HRSD-17) [35]. Exclusion criteria included: pregnant, breast feeding or planning to be pregnant during the course of trial, lifetime history of psychotic disorder or presence of current psychotic symptoms, diagnosis of anorexia or bulimia within last 2 years, current primary diagnosis of obsessive compulsive disorder, current substance dependence that requires inpatient detoxification or treatment, current psychiatric disorder that requires inpatient hospitalization, lifetime history or allergy to protocol antidepressants, history of non-response to antidepressant during current episode or non-response to protocol antidepressants ever in lifetime, taking any of the protocol antidepressants at screening visit, taking fluoxetine or monoamine oxidase inhibitors in 4 weeks prior to study entry, presence of unstable general medical condition that requires hospitalization, require medications for general medications that contraindicate protocol antidepressant, presence of epilepsy, lifetime history of seizure or other condition requiring anticonvulsant, presence of narrow angle glaucoma, hypothyroidism (unless stable on medication for last 3 months), and treatment with somatic antidepressants, contraindicated medications, or depression specific psychotherapy, also see the clinicaltrials.gov website (https://clinicaltrials.gov/ct2/show/NCT00590863). Consent was offered to 832 treatment-seeking depressed outpatients at participating clinical sites, of whom 731 were screened and 61 were excluded from randomization [9]. The target sample size for CO-MED trial was 660 to allow detection of 15% difference in remission rates between combinations of antidepressants and SSRI monotherapy [9].

The Institutional Review Boards at UT Southwestern Medical Center at Dallas, the University of Pittsburgh Data Coordinating Center, each participating regional center, and all relevant clinical sites reviewed and approved the study protocol and all consent documents and study procedures. All participants provided written informed consent prior to entering the study. An independent Data Safety and Monitoring Board also monitored the study. Further details of CO-MED study have been described by Rush et al. [9] (clinicaltrials.gov identifier NCT00590863).

### Medications

Participants in all three treatment-arms (SSRI monotherapy, bupropion-SSRI combination, and venlafaxine-mirtazapine combination) received two types of pills. The first medication was known to both participants and study personnel while the second medication was known only to the study personnel (single blind). All dose adjustments described below were made only if side effects were tolerable [9].

Participants in SSRI monotherapy treatment-arm were started on 10 mg/day dose of escitalopram with option to increase the dose to 20 mg/day at week 4 visit or later if QIDS-C score was greater than 5. Pill placebo was added at week 2 in single-blind fashion with option to increase the dose at week 4 visit or later if QIDS-C score was greater than 5. At the end of 12 weeks, mean escitalopram dose was 17.6 mg/day and mean placebo dose was 1.4 pills/day.

Participants in bupropion-SSRI combination treatment-arm were started on bupropion SR 150 mg/day and the dose was increased to 300 mg/day at week 1 visit. At week 2 visit, escitalopram 10 mg/day was started in single-blind fashion. At week 4 visit, bupropion SR was increased to 400 mg/day and/or escitalopram was increased to 20 mg/day if QIDS-C score was greater than 5. For visits at week 6 and later, doses could be increased to 400 mg/day of bupropion SR and 20 mg/day of escitalopram if QIDS-C score was greater than 5. At the end of 12 weeks, mean bupropion SR dose was 324.0 mg/day and mean escitalopram dose was 14.0 mg /day.

Participants in venlafaxine-mirtazapine combination treatment-arm were started on venlafaxine XR 37.5 mg/day for 3 days and then increased to 75 mg/day. At week 1 visit, venlafaxine XR was increased to 150 mg/day. At week 2 visit, if the score on QIDS-C was greater than 5 then mirtazapine 15 mg/day was added in single-blind fashion. At week 4 visit, if QIDS-C was greater than 5 then venlafaxine XR dose was increased to 225 mg/day and/or mirtazapine was increased to 30 mg/day. At week 6, if QIDS-C was greater than 5 then mirtazapine could be raised to 45 mg/day. At week 8, if QIDS was greater than 5 then venlafaxine XR could be raised to 300 mg/day. At the end of 12 weeks, mean venlafaxine XR dose was 207.6 mg/day and mean mirtazapine dose was 25.3 mg /day.

### Assessments

At baseline, participants provided sociodemographic information. At baseline and all treatment visits (weeks 1, 2, 4, 6, 8, 10, 12, 16, 20, 24, and 28), participants completed the 16-item Quick Inventory of Depressive Symptomatology—Self-Report (SR) scale [31, 36, 37] and the 5-item Work and Social Adjustment Scale (WSAS) [38] at identical assessment time points.

Quick Inventory of Depressive Symptomatology—Self-Report (QIDS-SR): Of the 16 items (each item has 4 choices which are scored from 0–3) in QIDS-SR, 9 items are used to calculate the total score which can range from 0–27 [31]. The Pearson moment correlation between QIDS-SR and HRSD-17 has been previously reported as 0.86 [36]. The Cronbach’s α of QIDS-SR ranged from 0.86–0.87 in previous reports [31, 36, 37].

Work and Social Adjustment Scale (WSAS): The WSAS is a 5-item self-report measure designed to identify functional impairment that is attributed to an identified problem or condition, and it has been used to study the treatment of depression and anxiety [38]. Each question is rated on a 0 to 8 scale, with 0 indicating no impairment, and 8 indicating very severe impairment. WSAS scores above 10 suggest significant functional impairment. Cronbach’s α measure of internal consistency ranges from 0.80 to 0.94, with test-retest reliability of 0.73. The WSAS also has good convergent and discriminant validity, and is sensitive to patient differences in severity, as well as treatment-related change [38, 39].

### Statistical analyses

Data from all 665 CO-MED participants were included in this report. To address these issues with replication of findings of model-based analyses, we decided to divide all CO-MED participants in two separate groups (model selection and validation samples) in a random fashion so as to draw inferences in the model selection sample and test for the consistency of these inferences in the validation sample. We compared baseline clinical and sociodemographic variables in both samples (model selection and validation) using chi-square test for categorical variables and analysis of variance (ANOVA) for categorical variables. To identify sub-groups of participants with different trajectories of changes in WSAS during the first 6 weeks of the trial, we used latent class growth analysis [40] using PROC TRAJ as implemented in SAS to estimate discrete mixture models with censored normal distribution [41, 42] in the model selection sample (maximum of 5 latent classes). We used the methods described in previous publications [25, 27, 43, 44] to select the final model based on model fit [Bayesian information criteria (BIC) and Akaike information criteria (AIC), with the lower values of BIC and AIC indicating better model fit [40]], parsimony (number of groups), membership size (percentage of sample in each trajectory group), and interpretability [statistical significance of modeling terms (for example, intercept only, one-, two-, three-, or four-degree polynomial)]. We then assigned participants in the validation sample to sub-groups based on trajectories of WSAS change previously estimated in the model selection sample. We calculated the estimated score for each trajectory using the following formula: (intercept) + (estimate of one-degree polynomial from final model selected in selection sample)*(week) + (estimate of two-degree polynomial from final model selected in selection sample)*(week)^**2**^ + (estimate of three-degree polynomial from final model selected in selection sample)*(week)^**3**^. For each individual participant, we used the observed score available at each visit and subtracted the estimated score of each trajectory from this observed score. We then squared the difference to calculate squared-error, giving us a squared-error value for each trajectory at each visit. We then calculated the mean of this squared-error across all visits for each trajectory in first 6 weeks for each participant and obtained a mean squared-error value for each trajectory. We assigned the participant in validation sample to the sub-group which had the trajectory with lowest mean squared-error.

Consistent with Rush et al.’s previous report of numerically lower values of WSAS at 3 and 7 months as compared to baseline [9], we hypothesized lower values at week 6 as compared to baseline. To evaluate whether reductions in WSAS during the first 6 weeks of treatment were statistically significant even after controlling for the select baseline covariates (listed below) and levels of QIDS-SR at each study visit, we used repeated measures mixed model analyses with visit as the within subject factor and all other variables as between subject factors. We calculated correlation coefficient between WSAS and QIDS using PROC MIXED as implemented in SAS [45] as we had repeated observations for each subject [46]. To evaluate whether participants in these sub-groups with different trajectories of WSAS change had different rates of remission, we used separate univariate logistic regression analyses followed by multivariate (select baseline covariates along with remission at week 6 and treatment-arm) analyses with backwards elimination, and remission at 3 and 7 months as dependent variables.

Based on literature review, we included the following baseline clinical and sociodemographic features as covariates for our analyses: age, education (< 12 years, 12–15 years, and ≥16 years), employment status (employed or unemployed), monthly income (< $2000, $2000 to $4000, and > $4000), gender, race (white, black, other), Hispanic ethnicity, anxious features, depression onset before age of 18, presence of suicidal ideations, QIDS-SR score, and WSAS score [14, 16, 47]. No interaction terms were included in the model. Even though treatment-arms do not differ as shown in the primary report on CO-MED [9], we included treatment-arm in our analyses to account for any variability due to treatment-arm assignment.

We set the threshold of significance at 0.05 and used SAS 9.3 (SAS Inc., Cary, NC) for all analyses.

## Results

We found no differences in the participants in model selection (n = 334) and validation (n = 331) samples (See Table 1). Using repeated observations of QIDS-SR and WSAS in both model selection and validation samples, we found that the correlation coefficient between WSAS and QIDS-SR was 0.69 in both samples.

**Table 1:** Baseline sociodemographic and clinical characteristics of participants in CO-MED trial.

	Model Selection Sample	Validation Sample		
Number	334	331		
Categorical variables	n (%)	n (%)	Chi-square*	p-value
Sex			2.25 (df = 1)	0.13
Male	116 (34.7%)	97 (29.3%)		
Female	218 (65.3%)	234 (70.7%)		
Race			0.09 (df = 2)	0.96
White	214 (64.1%)	211 (63.7%)		
Black	89 (26.6%)	87 (26.3%)		
Other	31 (9.3%)	33 (10%)		
Monthly income			0.30 (df = 2)	0.86
<$2000	189 (62%)	189 (63.6%)		
$2000 - $4000	65 (21.3%)	63 (21.2%)		
>$4000	51 (16.7%)	45 (15.2%)		
Treatment-arm			3.02 (df = 2)	0.22
SSRI monotherapy	123 (40.5%)	101 (30.5%)		
Bupropion-SSRI	107 (35.2%)	114 (34.4%)		
Venlafaxine-mirtazapine	104 (24.3%)	116 (35.1%)		
Education			1.23 (df = 2)	0.54
<12 years	44 (13.8%)	54 (16.8%)		
12–15 years	177 (55.3%)	174 (54.2%)		
≥16 years	99 (30.9%)	93 (29%)		
Hispanic ethnicity			0.14 (df = 1)	0.71
Yes	49 (14.7%)	52 (15.7%)		
No	285 (85.3%)	279 (84.3%)		
Employed			0.780 (df-1)	0.37
Yes	172 (51.50%)	159 (48.0%)		
No	162 (48.5%)	172 (52.0%)		
Anxious features present			0.37 (df = 1)	0.55
Yes	253 (76.0%)	244 (73.9%)		
No	80 (24.0%)	86 (26.1%)		
Suicidal ideation present			1.02 (df = 1)	0.31
Yes	192 (57.5%)	203 (61.3%)		
No	142 (42.5%)	128 (28.7%)		
Onset of depression before age 18			1.84 (df = 1)	0.17
Yes	140 (42.0%)	156 (47.3%)		
No	193 (58.0%)	174 (52.7%)		
Continuous variables	Mean (SD)	Mean (SD)	F value**	p-value
Age in years	42.44 (13.02)	42.98 (13.00)	0.29 (df = 1, 663)	0.59
QIDS-SR	15.36 (4.05)	15.53 (4.45)	0.29 (df = 1, 663)	0.59

All CO-MED trial participants (n = 665) were randomly divided in model selection and validation samples. CO-MED is Combining Medications to Enhance Depression Outcomes, n is number, SD is standard deviation, df is degrees of freedom, and QIDS-SR is Quick Inventory of Depressive Symptomatology—Self-Report, Selective Serotonin Reuptake Inhibitor (SSRI) refers to escitalopram, SSRI monotherapy is combination of escitalopram and placebo.

* Chi-square for categorical variable from chi-square test,

** F value for continuous variables from analysis of variance (ANOVA) test.

### Model development in the selection sample

As shown in Table 2, we systematically increased the number of classes and order of polynomials in the model selection sample and found that models with two sub-groups had the lowest BIC and AIC as compared to models with three or more sub-groups. We found that the model with best fit (lowest BIC and AIC) had two sub-groups with two- and zero-degree polynomials as order of changes in the trajectory. Next, to maximize the interpretability of our model, we examined the statistical significance of higher order polynomials in all two sub-group models, and found that the model with three- and one-degree polynomial order had significant p values for both these orders. Hence, we selected this model (three- and one-degree polynomial) over the model (two- and zero-degree polynomial) with lowest BIC and AIC in the model selection sample. See Table 3 for estimates of three- and one-degree polynomial parameters of the selected two sub-groups. Participants in the first sub-group (41.98%) had early improvement in WSAS whereas participants in the second sub-group (58.02%) experienced gradual change.

**Table 2:** Model fit estimates of latent class analyses of WSAS change in the model selection sample (n = 334).

Number of Sub-groups	Order of Polynomials	Bayesian information criteria (BIC)	Akaike information criteria (AIC)
2	1 1	-4921	-4909
	2 2	-4908	-4893
	3 3	-4905	-4886
	3 2	-4902	-4885
*Final model*	*3 1*	*-4900*	*-4885*
	2 1	-4906	-4892
	2 0	-4946	-4934
	3 0	-4942	-4929
3	3 3 3	-4847	-4818
	3 3 2	-4844	-4818
	3 3 1	-4841	-4817
	3 2 1	-4839	-4816
	2 2 2	-4851	-4828
	2 2 1	-4848	-4827
	1 1 1	-4865	-4848
4	3 3 3 3	-4834	-4796
	3 3 3 2	-4831	-4795
	3 3 3 1	-4828	-4794
	3 3 2 1	-4826	-4776
	3 3 1 1	-4823	-4793
	1 1 1 1	-4850	-4827
	2 2 2 2	-4835	-4804
	2 2 2 1	-4832	-4804
	2 2 1 1	-4829	-4803
5	3 3 3 3 3	-4834	-4876
	3 3 3 3 1	-4830	-4876
	3 3 3 2 1	-4825	-4783
	3 3 3 1 1	-4823	-4783
	3 3 2 1 1	-4820	-4782

WSAS is Work and Social Adjustment Scale, CO-MED is Combining Medications to Enhance Depression Outcomes. Please note that all AIC and BIC values are negative, hence higher numbers represent lower values.

**Table 3:** Parameters of selected model of change in WSAS during the first 6 weeks of CO-MED trial.

Sub-group (or trajectory)	Parameter	Estimate	Standard Error	t-value*	p value
1	Intercept	21.94	0.73	30.27	**<0.001**
	Linear	-10.34	1.24	-8.33	**<0.001**
	Quadratic	2.79	0.54	5.20	**<0.001**
	Cubic	-0.25	0.06	-4.20	**<0.001**
2	Intercept	31.13	0.47	66.58	**<0.001**
	Linear	-1.40	0.14	-9.90	**<0.001**
	Sigma	8.03	0.17	48.09	**<0.001**
1	Membership (%)	41.98	3.20	13.12	**<0.001**
2	Membership (%)	58.02	3.20	18.13	**<0.001**

Bayesian Information Criteria (BIC) = -4900; Akaike Information Criteria (AIC) = -4885. These parameters are from the final selected 2-class 3-degree censored normal model of changes in Work and Social Adjustment Scale (WSAS) from baseline to week 6 in the model selection sample (n = 334) obtained after randomly dividing all participants (n = 665) of the Combining Medications to Enhance Depression Outcomes (CO-MED) trial in two halves.

*The t-value is for null hypothesis that estimate for each parameter equals 0. The BIC and AIC are overall model fit estimates.

### Improvements in psychosocial function during first six weeks of antidepressant treatment

We found that reduction in WSAS from baseline to week 6 were significant in the validation sample (F = 6.75 df = 3, p <0.001) even after controlling for QIDS-SR at each visit and adjusting for above-mentioned baseline clinical and sociodemographic variables in repeated measures mixed model analyses (also see S1 Table for results of mixed model analyses in both model selection and validation samples).

### Trajectories of changes in WSAS during the first 6 weeks in the validation sample

**Fig 1:**
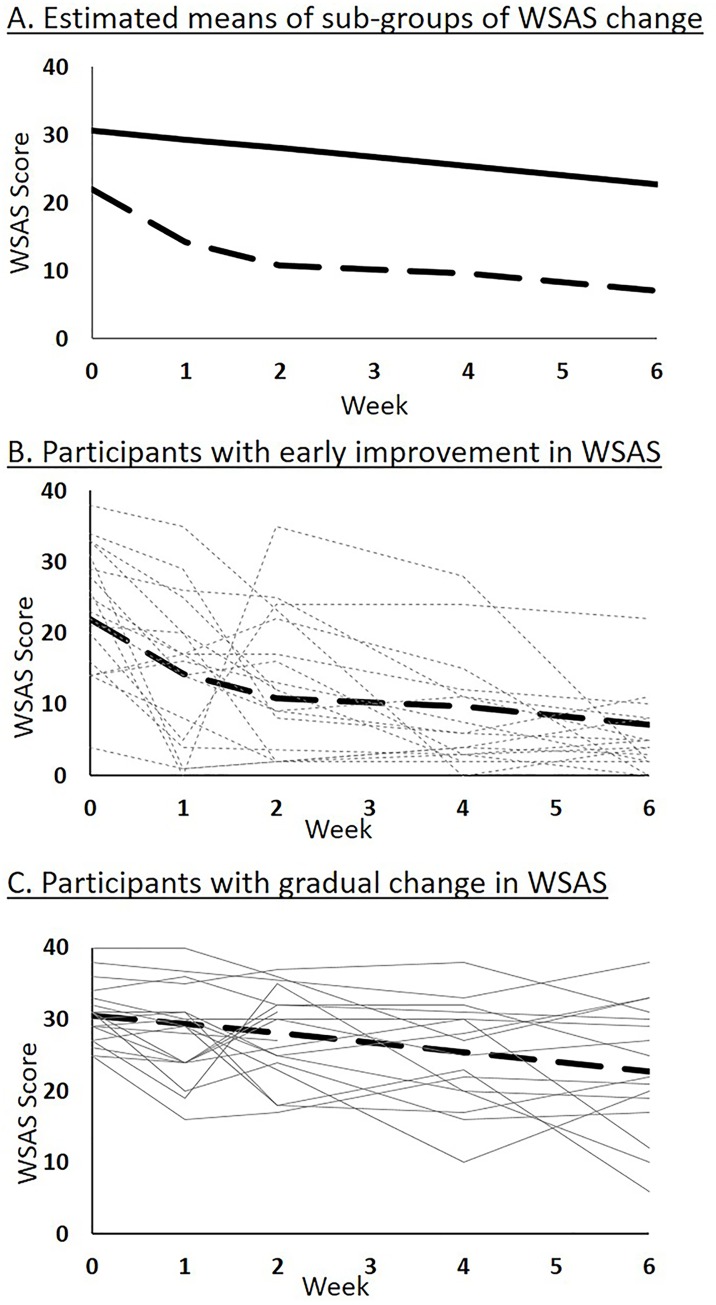
The final two sub-group model of WSAS change during first 6 weeks of the CO-MED trial. A. Estimated means of the two sub-groups, gradual change (solid line) and early improvement (broken line or dash), identified by latent class analysis of Work and Social Adjustment Scale (WSAS) changes during the first 6 weeks of the Combining Medications to Enhance Depression Outcomes (CO-MED) trial. B. Estimated mean along with observed values of 20 randomly selected participants from early improvement sub-group of the validation sample (n = 331). C. Estimated mean along with observed values of 20 randomly selected participants from gradual change sub-group of the validation sample (n = 331).

**Fig 2:**
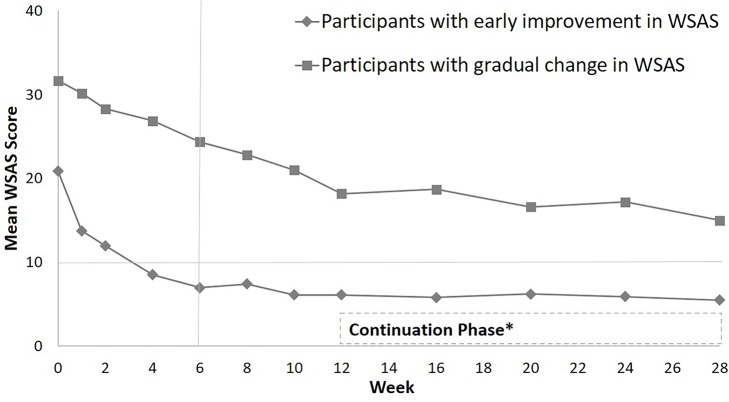
Psychosocial function of validation sample participants (n = 331) during CO-MED trial. Sub-groups (early improvement and gradual change) of participants with different trajectories of Work and Social Adjustment Scale (WSAS) change were identified using latent class analyses during the first 6 weeks (marked by vertical line). Score of WSAS greater than 10 suggest significant functional impairment [38] (horizontal line). * Continuation Phase was limited to CO-MED trial participants who had a clinical response.

In the validation sample, 46.83% exhibited early improvement in WSAS whereas 53.17% experienced gradual change. The estimated means for the two sub-groups generated by PROC TRAJ procedure are presented in panel A of Fig 1. Even though the participants with early improvement had on average lower baseline WSAS scores, there was substantial overlap in WSAS scores between the two sub-groups at baseline but from week 2 onwards the two sub-groups sharply diverged; see panels B and C of Fig 1, which present observations from 20 randomly selected participants from each sub-group in the validation sample. From baseline to week 6, participants with early improvement had significantly lower WSAS as compared to those with gradual change (estimate = 6.9, standard error = 1.1) with an effect size of 0.69. We have presented the mean WSAS scores for the two sub-groups in Fig 2 to depict the course of change in psychosocial function during the CO-MED trial.

### Remission rates at 3 and 7 months in the validation sample based on trajectories of WSAS change

As compared to participants with gradual change in WSAS, those with early improvement had significantly higher unadjusted rates of remission at both 3 months (OR = 4.94; 95% CI = 2.78,8.80) and 7 months (OR = 3.30; 95% CI = 2.14,5.08). Even after controlling for the above-mentioned baseline variables and remission status at week 6, rates of remission in participants with early improvement of WSAS continued to be higher at both 3 months (OR = 3.29; 95% CI = 1.65,6.52) and 7 months (OR = 2.31; 95% CI = 1.37,3.90) as compared to those with gradual change. These findings were similar to those in the model selection sample, see S2 Table.

## Discussion

To the best of our knowledge, this is the first report that used randomly selected model selection and validation samples to assess and replicate the prognostication of longer-term clinical outcomes based on early changes in psychosocial function in a large cohort of MDD patients treated with antidepressant medications. We found that psychosocial function, as measured by WSAS, improved significantly during the first 6 weeks of antidepressant treatment, independent of change in depressive symptom severity. We also found that when divided into two distinct sub-groups based on trajectories of change in psychosocial function during first 6 weeks of antidepressant treatment (early improvement and gradual change), participants with early improvement in psychosocial function were 3–6 times more likely to be in remission at 3 months and 2–3 times more likely at 7 months, even after controlling for remission status at week 6, baseline symptom severity, and other variables.

These findings extend previous studies reporting substantial improvement in psychosocial function with a variety of treatments [7–13, 48]. They also add to an emerging literature that suggests that at least a subgroup of depressed individuals realize functional improvements early in the course of antidepressant treatment, and these early changes in work productivity and psychosocial function predict future clinical course [27]. Our finding that change in depression severity did not fully account for improved psychosocial function supports the notion that burden of depressive illness and improvement with treatment are not just restricted to core depressive symptoms [5]. These results also suggest the need to direct additional treatment modalities for those with inadequate psychosocial improvement early in the course of treatment, as well as routine measurement of psychosocial function to ensure the goal of recovery is achieved.

Strengths of our study include the adaptation of the commonly used data-driven trajectory-based approach using latent class analyses [49–51] to identify sub-groups of MDD patients with different patterns (linear or non-linear) of changes in psychosocial function that can be missed by traditional linear mixed model analyses [52]. We have previously used a similar data-driven approach to identify sub-groups of participants with different trajectories of change in work productivity outcomes (absence from work and/or reduced productivity while at work) [27] for those who were employed. In the context of model-based analysis to understand the relationship of psychosocial function and depression severity, two recent studies that used structural equation modeling (SEM) had conflicting findings. While Dunn et al. found that improvement in psychosocial function predicted subsequent improvement in depression severity [17], Lin et al. failed to replicate this finding using similar SEM analyses [53]. To our knowledge, no one has previously either tried to replicate findings from latent class analysis of longitudinal data or address the limitation of model-overfitting. Hence, we designed our analyses to be conducted first in a model selection sample and then be repeated in a validation sample. We divided the sample randomly to ensure that both samples (model selection and validation) were similar on baseline sociodemographic and illness characteristics. We developed data-driven trajectory models using latent class analyses in the model selection sample, selected a final model, and assigned participants in the validation sample to sub-groups with the same mean estimates of WSAS change as those in the final selected model of the model selection sample. Our method of assigning sub-groups based on different trajectories of WSAS change in the validation sample, which uses a simple mathematical equation along with the model-estimates from the model selection sample, is easy to use in future studies. This is highly significant in light of widespread attention on non-replication of previously reported findings [29]. Hence, the consistent finding of higher remission rates at 3 and 7 months based on early improvement in psychosocial function in both the model selection and validation samples is a major strength of our study.

### Study limitations

Our study has several limitations. The analyses conducted in this report are secondary in nature. The model developed and validated in this report was based on participants from a clinical trial, though one with broad inclusion and minimal exclusion criteria. However, despite the fact that CO-MED participants were recruited from real-world outpatient clinics with broad inclusion criteria, treatment was delivered using high quality MBC as outlined by Trivedi et al. [2]. Future studies that prospectively identify trajectories of change in psychosocial function and their association with different remission rates would further support our findings. However, we have outlined how one can replicate these findings to see if such an approach is viable. The measure of psychosocial function in our report, WSAS, is a brief instrument which may not comprehensively evaluate all dimensions of psychosocial function that may be relevant to assessing functional recovery.

## Conclusions

In conclusion, we found that psychosocial function, as captured by the WSAS, is not merely a proxy of depressive symptom severity and can improve early in the course of antidepressant treatment. Those MDD patients who have early improvement of psychosocial function have higher rates of longer-term remission (3 and 7 months) than those with gradual change.

## Supporting Information

S1 Table Results of mixed model analyses with WSAS at week 6 as the dependent variable. (DOCX)

S2 Table Prediction of longer-term remission based on changes in WSAS in model selection sample (n = 334). (DOCX)
