# Supplementary material for: Early Improvement in Psychosocial Function Predicts Longer-Term Symptomatic Remission in Depressed Patients
Source: PLoS One. 2016 Dec 28;11(12):e0167901. doi: 10.1371/journal.pone.0167901 (PMC5193346; doi:10.1371/journal.pone.0167901)
Supplement: S1 Table — (DOCX) [file pone.0167901.s001.docx]

S1 Table. Results of mixed model analyses with WSAS at week 6 as the dependent variable

|  | Model Selection Sample (n= 334) | | Validation Sample (n = 331) | |
| --- | --- | --- | --- | --- |
|  | F value (df) | p Value | F value (df) | p Value |
| *Time* | *8.15 (3)* | *<0.001* | *6.75 (3)* | *<0.001* |
| QIDS-SR at each visit* | 365.92 (1) | <0.001 | 309.88 (1) | <0.001 |
| Baseline QIDS-SR | 2.72 (1) | 0.10 | 0.14 (1) | 0.70 |
| Baseline WSAS | 90.36 (1) | <0.001 | 86.66 (1) | <0.001 |
| Age (in years) | 7.45 (1) | 0.007 | 2.18 (1) | 0.14 |
| Hispanic ethnicity | 1.39 (1) | 0.24 | 0.84 (1) | 0.36 |
| Race | 5.28 (2) | 0.006 | 1.05 (2) | 0.35 |
| Treatment-arm | 0.41 (2) | 0.66 | 0.56 (2) | 0.57 |
| Gender | 1.16 (1) | 0.27 | 0.23 (1) | 0.64 |
| Anxious features at baseline | 0.40 (1) | 0.53 | 0.21 (1) | 0.65 |
| Depression onset before age 18 | 0.54 (1) | 0.46 | 0.79 (1) | 0.37 |
| Employment | 6.77 (1) | 0.01 | 8.19 (1) | 0.005 |
| Education | 0.04 (2) | 0.96 | 0.09 (2) | 0.91 |
| Income | 1.16 (2) | 0.31 | 0.45 (2) | 0.64 |
| Suicidal ideations at baseline | 0.53 (1) | 0.47 | 1.25 (1) | 0.27 |

All CO-MED trial participants (n=665) were randomly divided into model selection and validation samples, and separate repeated-measures mixed model analyses for these samples were conducted with WSAS as the dependent variable. * Does not include baseline QIDS-SR, df is degrees of freedom, WSAS is Work and Social Adjustment Scale, and QIDS-SR is Quick Inventory of Depressive Symptomatology Self-Report. Race variable includes white, black, and other; treatment-arm includes escitalopram plus placebo, sustained-release bupropion plus escitalopram, and extended-release venlafaxine plus mirtazapine; education includes less than 12 years, 12 to 15 years, and greater than or equal to 16 years; income includes monthly income of less than $2000, $2000 - $4000, and greater than $4000.
