# Supplementary material for: Early Improvement in Psychosocial Function Predicts Longer-Term Symptomatic Remission in Depressed Patients
Source: PLoS One. 2016 Dec 28;11(12):e0167901. doi: 10.1371/journal.pone.0167901 (PMC5193346; doi:10.1371/journal.pone.0167901)
Supplement: S2 Table — (DOCX) [file pone.0167901.s002.docx]

S2 Table. Prediction of longer-term remission based on changes in WSAS in model selection sample (n=334).

|  | Remission at 3 months | | Remission at 7 months | |
| --- | --- | --- | --- | --- |
|  | Odds Ratio | 95% CI | Odds Ratio | 95% CI |
| Unadjusted - univariate logistic regression analysis |  |  |  |  |
| Early improvement vs. gradual change | 9.87 | 5.10,19.10 | 3.39 | 1.79,6.42 |
| Adjusted* - multivariate logistic regression analysis |  |  |  |  |
| Early improvement vs. gradual change | 5.61 | 2.72,11.57 | 3.04 | 1.60,5.79 |

* Adjusted for baseline covariates (age, gender, race, ethnicity, education, income, employment status, depression onset before age 18, anxious features, suicidal ideation, treatment-arm, and WSAS scores) as well as remission status at week 6. All model fits were found to be adequate (Hosmer and Lemeshow test p-value > 0.10).
